# Effects of a novel synbiotics-enzyme complex as a replacement for antibiotics on growth performance, slaughter and meat characteristics, immune organ index, and intestinal morphology of broilers

**DOI:** 10.3389/fvets.2024.1468847

**Published:** 2024-10-17

**Authors:** Zihao Zhao, Simushi Liswaniso, Ning Qin, Shengxiao Cao, Xin Wu, Chang Ma, Chunchi Yan, Rifu Xu, Xue Sun

**Affiliations:** ^1^College of Animal Science and Technology, Jilin Agricultural University, Changchun, China; ^2^Joint International Research Laboratory of Modern Agricultural Technology, Ministry of Education, Jilin Agricultural University, Changchun, China

**Keywords:** AA+ broiler, linear body measurements, meat quality, prebiotics, probiotics, synbiotics

## Abstract

**Introduction:**

Antibiotic use in broilers is being discouraged globally due to the challenges it poses. This study was conducted to assess the effects of supplementing broilers with a Symbiotic-Enzyme complex (SEC) containing prebiotics (*mannose oligosaccharides*), probiotics (*Clostridium butyricum* and *Bacillus subtilis*), and enzymes (*glucose oxidase*, and α*-galactosidase*) as an alternative to antibiotics on growth performance, carcass and meat quality traits, mortality, linear body measurements, intestinal morphology and immune organ indexes.

**Method:**

A total of 864 mixed-sex 1-day-old arbor acres (AA+) broilers were allocated to 8 experimental groups replicated 9 times with 12 chickens per replicate. These included 6 treatment groups with SEC inclusion levels of 0.025, 0.04, 0.05, 0.06, 0.08, and 0.10%, respectively, and two control groups: a negative control group containing a basal diet only and the positive control group (Antibiotics group) containing a basal diet and antibiotic oxytetracycline added at 0.2%. Growth performance was measured on day 21 and 42, and the mortality, carcass, meat quality traits, linear body measurements, intestinal morphology, and organ size indexes were measured on day 42.

**Results:**

The results indicated that supplementing broilers with 0.1% SEC resulted in insignificant (*P* > 0.05) increases in average daily feed intake (ADFI), significant (*P* < 0.05) increases in the average daily gains (ADG), and significant (*P* < 0.05) reduction in a feed-to-gain ratio (F/G) in all the phases compared to the control and antibiotics groups. Supplementation of broilers with 0.1% SEC inclusion levels also significantly (*P* < 0.05) increased the body slope length, chest width, chest depth, keel length, and shank circumference. Furthermore, broilers on diets containing 0.1% SEC inclusion level also had significantly (*P* < 0.05) higher dressed, semi-evisceration, evisceration, and breast muscle percentages. Including SEC at 0.1% also significantly (*P* < 0.05) increased villus height and villus-to-crypt ratio (V/C) but reduced crypt depth in the duodenum, jejunum, and ileum compared to the control groups. SEC inclusion at 0.1% significantly (*P* < 0.05) increased the spleen, bursal, and thymus indexes, respectively.

**Conclusion:**

Supplementation of broilers with 0.1% SEC can be used as an antibiotic alternative because it increases the F/G, improves the carcass and meat quality, increases the body conformation, improves the small intestines' functions, and immune organ size.

## 1 Introduction

Antibiotics in broilers have been used to enhance the production and productivity of chickens by controlling or preventing the growth of pathogenic bacteria, promoting enhanced immune function, and improving gut health, thus enhancing feed efficiency (1). However, misuse of these antibiotics can lead to challenges of antimicrobial resistance (2). As such, the last few years have seen a ban on the use of antibiotics in poultry production mainly due to the concerns of antibiotic residues leading to antibiotic resistance. Specifically, the European Union is among the zones where the use of antibiotics as growth promoters was banned (3). This has led to researchers to find possible alternatives to antibiotics as growth promoters without compromising the quality of the production and productivity of broilers that may be affected by this ban.

Probiotics, prebiotics, and some enzymes have been identified as suitable antibiotic substitutes. Probiotics support immune system function, balance intestinal microbiota, improve growth and nutritional digestibility, and improve intestinal morphology in the host animal (4–6). *Clostridium butyricum* and *Bacillus subtilis* are among the probiotics being extensively tested in broilers (7). Prebiotics are non-digestible substances, mainly oligosaccharides, that stimulate the growth of beneficial bacteria in the gastrointestinal tract, such as lactobacilli and bifidobacteria. They also reduce the presence of harmful bacteria by competing for attachment sites on the intestinal lining (8). *Mannose oligosaccharides* are an example of some prebiotics presently under consideration as substitutes for antibiotics in broilers (9). However, a combination of prebiotics and probiotics, usually known as synbiotics, have also been tried and recommended (10). Enzymes such as *glucose oxidase* (GOD) and α*-galactosidase* (AlphaGal) have also been considered potential growth promoters in broilers (11, 12).

According to the International Scientific Association for Probiotics and Prebiotics (ISAPP), synbiotics are a blend of live microorganisms (probiotics) that are used explicitly by beneficial microorganisms within the host, leading to health benefits (13). This combination also includes a substrate (prebiotics) that is selectively utilized by the host's microorganisms, providing additional health advantages (14). Incorporating synbiotics as a feed supplement may enhance feed efficiency more effectively than using prebiotics and probiotics individually (15). Several studies have focused on using probiotics and prebiotics singularly in broilers, trying out several combinations. However, the combination of *Clostridium butyricum, Bacillus subtilis, Mannose oligosaccharides, glucose oxidase (GOD)*, and α*-galactosidase* (AlphaGal) has yet to be exploited in broilers as an alternative to antibiotics as promoting growth. Therefore, we hypothesized that this combination would positively work as an alternative to antibiotics as growth promoters and improve broilers' performance, meat quality, and immunity. Therefore, this study aimed to evaluate the effect of combining prebiotics (*mannose oligosaccharides*), probiotics (*Clostridium butyricum* and *Bacillus subtilis*) and enzymes [*glucose oxidase (GOD*), and α-*galactosidase* (AlphaGal)] as an alternative to antibiotics as growth promoters.

## 2 Materials and methods

### 2.1 Birds, diets, and management

A total of 864 mixed-sex 1-day-old arbor acres (AA+) broilers were used for this experiment. They were randomly assigned to eight groups replicated 9 times with 12 birds per replicate. The groups included the control group (negative control), the antibiotics group (positive control), and six (6) groups that contained different addition levels of the SEC that comprised *Clostridium butyricum, Bacillus, manno-oligosaccharides, glucose oxidase* and α*-galactosidase* in the ratio 1:10:3:3:3. The control group was only fed a basal diet meeting the nutritional requirements. Another group had the basal diet with an antibiotic (oxytetracycline) added at 0.2% (positive control). The treatment groups had 0.025, 0.04, 0.05, 0.06, 0.08, and 0.1% SEC inclusion levels designated E1, E2, E3, E4, E5, and E6 respectively (Table 1). The basal diets used in the entire study period are shown in Table 2.

**Table 1:** SEC supplementation in broiler diets.

**Group**	**Treatment**
Control group (C)	Basic diet only
Antibiotic group (A)	Basic diet + 0.2% oxytetracycline
Group 1 (E1)	Basic diet + 0.025% SEC
Group 2 (E2)	Basic diet + 0.04% SEC
Group 3 (E3)	Basic diet + 0.05% SEC
Group 4 (E4)	Basic diet + 0.06% SEC
Group 5 (E5)	Basic diet + 0.08% SEC
Group 6 (E6)	Basic diet + 0.1% SEC

SEC, synbiotics enzyme complex containing; *Clostridium butyricum*, Bacillus preparations, manno-oligosaccharides, glucose oxidase and α-galactosidase was 1:10:3:3:3.

**Table 2:** Basal diets for broilers during the 42 days growth period.

**Item**	**1–14 days**	**15–28 days**	**29–42 days**
**Composition of basic diet, %**			
Corn	57.8	54.3	59.23
Soybean meal (crude protein 43%)	28.7	30.2	22.48
Corn gluten meal	7.1	5	5.13
Feather meal	0	0	2.05
Limestone	1.4	1.2	1.13
Dicalcium phosphate	1.6	1.1	1.13
Soybean oil	1.6	6.5	7.51
Sodium chloride	0.2	0.3	0.3
Premix	1.6	1.4	1.04
Total	100	100	100
**Nutritional levels**			
Metabolizable energy (MJ/kg)	12.54	13.59	14.63
Crude protein, %	22.25	21.5	20
Lysine, %	1	0.7	2
Methionine + threonine, %	0.45	0.31	0.54
Calcium, %	1.01	0.83	0.77
Available phosphorus, %	0.33	0.34	0.35
Total phosphorus, %	0.64	0.55	0.52

Premix per kilogram of compound feedcontained: Vitamin A 18,000 IU, Vitamin D3 5,625 IU, Vitamin E 95 IU, Vitamin K 4.5 mg, Vitamin B1 3.6 mg, Vitamin B2 11.25 mg, Vitamin B12 45 μg, Niacinamide 72 mg, Pantothenic Acid 22.5 mg, Biotin 0.27 mg, Folic Acid 2.25 mg, Iron 60 mg, Copper 8 mg, Manganese 100 mg, Zinc 80 mg, Iodine 0.7 mg, Selenium 0.35 mg.

Metabolizable energy is a calculated value, while the other nutritional levels are measured values.

Before the experiment, thorough cleaning and disinfection of the chicken house were carried out to maintain a clean environment. The AA+ broiler chickens were raised in three-tier cages, each replicate in one cage. The birds had free access to feed and water, and proper ventilation was ensured in the chicken house. Feed was added and recorded twice daily, in the morning and evening, starting from 1 day old until the final day (day 42). Birds were vaccinated against known diseases as per the standard procedure.

### 2.2 Growth performance

AA+ broilers were weighed on an empty stomach on days 7, 14, 21, 28, 35, and 42 of age. The average daily weight gain (ADG) of AA+ broilers in each group was recorded at 7, 14, 21, 28, 35, and 42 days of age. Feed intake (FI) was recorded daily and aggregated weekly. The feed-to-gain ratio (F/G) was taken as the feed consumed as a fraction of the weight gain. On day 42 of the experiment, the best-performing treatment group among the treatment groups (with better ADFI, ADG, and F/G) was identified and enrolled for subsequent experiments.

### 2.3 Linear body measurements

On day 42, 60 AA+ broilers were randomly selected from the control group, antibiotic group, and best-performing treatment group (E6), respectively, to measure their body length, chest depth, chest width, keel length, thigh length, and thigh circumference as done according to the methods published previous (16, 17).

### 2.4 Slaughter performance

On 42-day, 60 AA+ broilers selected from each of the control group, antibiotic group, and best-performing treatment group were slaughtered by cutting the neck arteries, followed by scalding in hot water for feather removal. The post-slaughter body weight, whole leg weight, thigh weight, breast muscle weight, wing weight, abdominal fat weight, semi-eviscerated weight, and eviscerated weight, were measured. The dressed percentage, semi-eviscerated percentage, eviscerated percentage, breast muscle percentage, leg muscle percentage, and abdominal fat percentage were then calculated, respectively.

### 2.5 Immune organ index

From the selected 60 AA+ broilers from each group (control, antibiotics, and E6) the bursa, thymus, and spleen were isolated, blotted with filter paper to remove blood stains, and weighed after the removal of surface fat and connective tissue, and the immune organ index was calculated as follows:


$$\begin{array}{l} ImmuneOrganIndex=\frac{fresh\;weight\;of\;organ\;\left(g\right)}{live\;weight\;before\;slaughter}\left(kg\right) \end{array}$$

### 2.6 Meat quality

#### 2.6.1 Meat color

At 45 min after slaughter, a tristimulus colorimeter (CR-410) was used to measure the brightness (L^*^), redness (a^*^), and yellowness (b^*^) of the breast and thigh muscles of the selected 60 AA+ broilers from each control, antibiotics, and best-performing group (E6).

#### 2.6.2 pH value

After 45 min post broiler slaughter, a calibrated needle-type pH meter (HI-9024) was fully inserted into samples of the breast and thigh muscles of the broilers, and the pH values were recorded after 24 h.

#### 2.6.3 Shear force

After 48 h post-slaughter, respective muscle samples were stored for maturation. Subsequently, they were placed at room temperature, and a thermometer was inserted into the muscle near the central position. Then, the muscle was heated in a constant water bath at 70°C. Heating was stopped when the thermometer reading reached 60°C, cooled to room temperature, the muscle samples were removed, surface moisture was absorbed with filter paper, and then the muscle samples were cut into regular strips. Under room temperature conditions, the shear force of the long muscle strips was measured using a C-LM3B digital muscle tenderness meter, and the shear pressure displayed on the monitor was recorded.

### 2.7 Intestinal tissue morphology

Dissection was performed on 60 AA+ broilers selected from the control, antibiotic, and best-performing treatment groups (E6). Intestinal tissues from the duodenum, jejunum, and ileum were washed with 0.9% physiological saline, fixed in a 4% paraformaldehyde solution, and trimmed, rinsed, dehydrated, clarified, embedded, sectioned, and stained with routine hematoxylin-eosin (HE). Following the preparation of intestinal tissue sections, we observed and captured images using an optical microscope. We measured the villus height (VH) and crypt depth (CD) of the duodenum, jejunum, and ileum tissues using Image-ProPlus6.0 image analysis software. We then calculated the ratio of villus height to crypt depth (V/C).

### 2.8 Statistical analysis

The experimental data were inputted and processed in Excel 2016. One-way analysis of variance (ANOVA) was employed to evaluate and compare the means across the groups, while Tukey's *post-hoc* test was utilized to ascertain the significance of the observed differences. The results were presented as mean ± the standard deviation (SD). Significance was attributed to *P*-values <0.05.

## 3 Results

### 3.1 Performance, body morphology, and carcass traits

Table 3 shows the effect of supplementing broilers with Synbiotics-Enzyme Complex (SEC) on average daily weight gain (ADG), average daily feed intake (ADFI), and feed-to-gain ratio (F/G) over the 42-day production cycle. In all the growth phases, 1–21 d, 22–42 d, and 1–42 d, ADFI did not differ significantly (*P* > 0.05) between the groups. Between 1 and 21 days period, there were significant (*P* < 0.05) differences in ADG. As the amount of SEC increased, ADG also increased linearly, with the E6 group having the highest ADG. Between 22 and 42 d, there were significant (*P* < 0.05) differences in ADG between the groups. However, the E6 group was insignificantly higher (*P* > 0.05) than the control group but significantly higher (*P* < 0.05) than the antibiotics group. Overall (1–42 d), supplementation of broilers with SEC linearly increased their ADG, with the E6 groups having a significantly (*P* < 0.05) highest ADG.

**Table 3:** Effects of synbiotics enzyme complex on growth performance of broilers.

**Item**	**Control group**	**Antibiotics group**	**Dietary synbiotics enzyme complex level**					
			**E1**	**E2**	**E3**	**E4**	**E5**	**E6**
**Days 1–21**								
ADFI	57.69 ± 2.12	58.03 ± 3.04	60.08 ± 1.11	60.47 ± 1.85	59.81 ± 1.36	59.65 ± 2.86	60.47 ± 2.17	61.06 ± 3.14
ADG	41.60 ± 3.83^b^	42.11 ± 3.69^b^	43.36 ± 6.00^ab^	43.78 ± 2.98^ab^	44.14 ± 3.66^ab^	43.72 ± 5.54^ab^	44.84 ± 3.71^ab^	45.74 ± 5.37^a^
F/G	1.39 ± 0.02^a^	1.38 ± 0.02^a^	1.38 ± 0.01^a^	1.38 ± 0.01^a^	1.35 ± 0.01^bc^	1.36 ± 0.01^b^	1.34 ± 0.01^cd^	1.33 ± 0.01^d^
**Days 22–42**								
ADFI	160.93 ± 6.71	163.93 ± 4.84	158.91 ± 3.23	156.20 ± 10.53	158.46 ± 7.68	159.59 ± 10.71	158.24 ± 3.90	158.67 ± 3.31
ADG	87.44 ± 8.27^ab^	86.15 ± 15.28^b^	85.26 ± 7.14^b^	86.22 ± 5.87^b^	89.30 ± 8.50^ab^	89.94 ± 8.87^ab^	89.99 ± 8.73^ab^	94.25 ± 13.58^a^
F/G	1.82 ± 0.04^a^	1.80 ± 0.05^b^	1.82 ± 0.02^a^	1.81 ± 0.01^ab^	1.78 ± 0.01^c^	1.77 ± 0.01^c^	1.76 ± 0.01^c^	1.75 ± 0.01^c^
**Days 1–42**								
ADFI	104.21 ± 2.65	105.83 ± 3.38	104.69 ± 1.93	103.77 ± 3.64	104.40 ± 2.58	104.89 ± 3.12	104.73 ± 2.44	105.16 ± 2.87
ADG	64.52 ± 4.56^b^	64.13 ± 7.27^b^	64.31 ± 3.43^b^	65.00 ± 3.57^b^	66.72 ± 4.39^ab^	66.83 ± 4.47^ab^	67.42 ± 3.64^ab^	69.99 ± 6.00^a^
F/G	1.61 ± 0.04^a^	1.59 ± 0.05^ab^	1.60 ± 0.01^a^	1.60 ± 0.01^a^	1.56 ± 0.01^cd^	1.57 ± 0.01^bc^	1.55 ± 0.01^cd^	1.54 ± 0.02^d^

Data in the same column with different shoulder-marked letters indicate significant differences (*P* < 0.05), while the same letters or no letters indicate non-significant differences (*P* > 0.05). ADFI, average daily feed intake; ADG, average daily gain; F/G, feed to gain ratio.

Adding SEC to broiler feed significantly (*P* < 0.05) lowered the F/G from day 1 to day 21 compared to the control and antibiotic groups (Table 1). The E6 group had the lowest F/G. During 22–42 d, the E3, E4, E5, and E6 groups had significantly (*P* < 0.05) lower F/G ratios compared to the control and antibiotic groups. However, groups E1 and E2 did not differ significantly (*P* > 0.05) from the control and antibiotic groups. Nevertheless, during the entire production period (1–42 d), F/G significantly (*P* < 0.05) decreased in groups E3, E4, E5, and E6 compared to the control and the antibiotics groups, with the E6 group having the lowest F/G ratio. During the period 1–42 d, E1 and E2 did not differ significantly (*P* > 0.05) from the control and antibiotic groups. We then selected the E6 group, here further referred to as the SEC group, to proceed with the study owing to its superior performance relative to the other treatment groups.

Table 4 shows the effect of SEC supplementation on broiler body morphometry. The SEC-supplemented group had the highest measures of all the body measurements taken. SEC supplementation significantly (*P* < 0.05) increased body slope length compared to the control and the antibiotic groups. The SEC group exhibited significantly (*P* < 0.05) greater chest width, chest depth, keel length, and shank circumference than the control. However, these measurements were insignificantly (*P* > 0.05) higher than those of the antibiotic groups. The SEC supplementation had no impact on the shank length.

**Table 4:** Effects of the synbiotics-enzyme complex on linear body measurements.

**Linear body measurements**	**Control group**	**Antibiotics group**	**E6**
Body slope length (cm)	22.42 ± 0.89^c^	23.38 ± 1.03^b^	23.94 ± 1.01^a^
Chest width (cm)	10.23 ± 0.66^b^	10.70 ± 0.60^a^	10.70 ± 0.73^a^
Chest depth (cm)	14.05 ± 0.62^b^	14.33 ± 0.60^a^	14.41 ± 0.64^a^
Keel length (cm)	14.26 ± 0.63^b^	14.44 ± 0.74^ab^	14.60 ± 0.60^a^
Shank length (cm)	7.96 ± 0.40	8.06 ± 0.45	8.07 ± 0.51
Shank circumference (cm)	5.32 ± 0.48^b^	5.41 ± 0.41^ab^	5.51 ± 0.46^a^

Data in the same column with different shoulder-marked letters indicate significant differences (*P* < 0.05), while the same letters or no letters indicate non-significant differences (*P* > 0.05).

**Figure 1:**
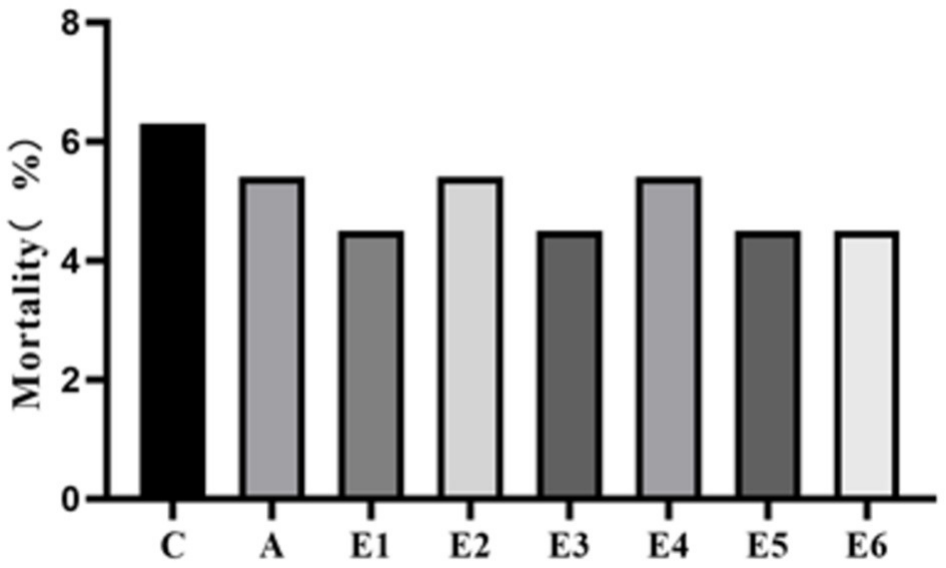
Effects of synbiotics enzyme complex on the mortality of broilers.

Supplementation of feed with SEC did not affect the mortality of the broilers (*P* > 0.05) (Figure 1). The control groups had insignificantly (*P* > 0.05) higher mortality rates than the rest, suggesting that mortality may not be associated with the treatments (SEC supplementation).

**Figure 2:**
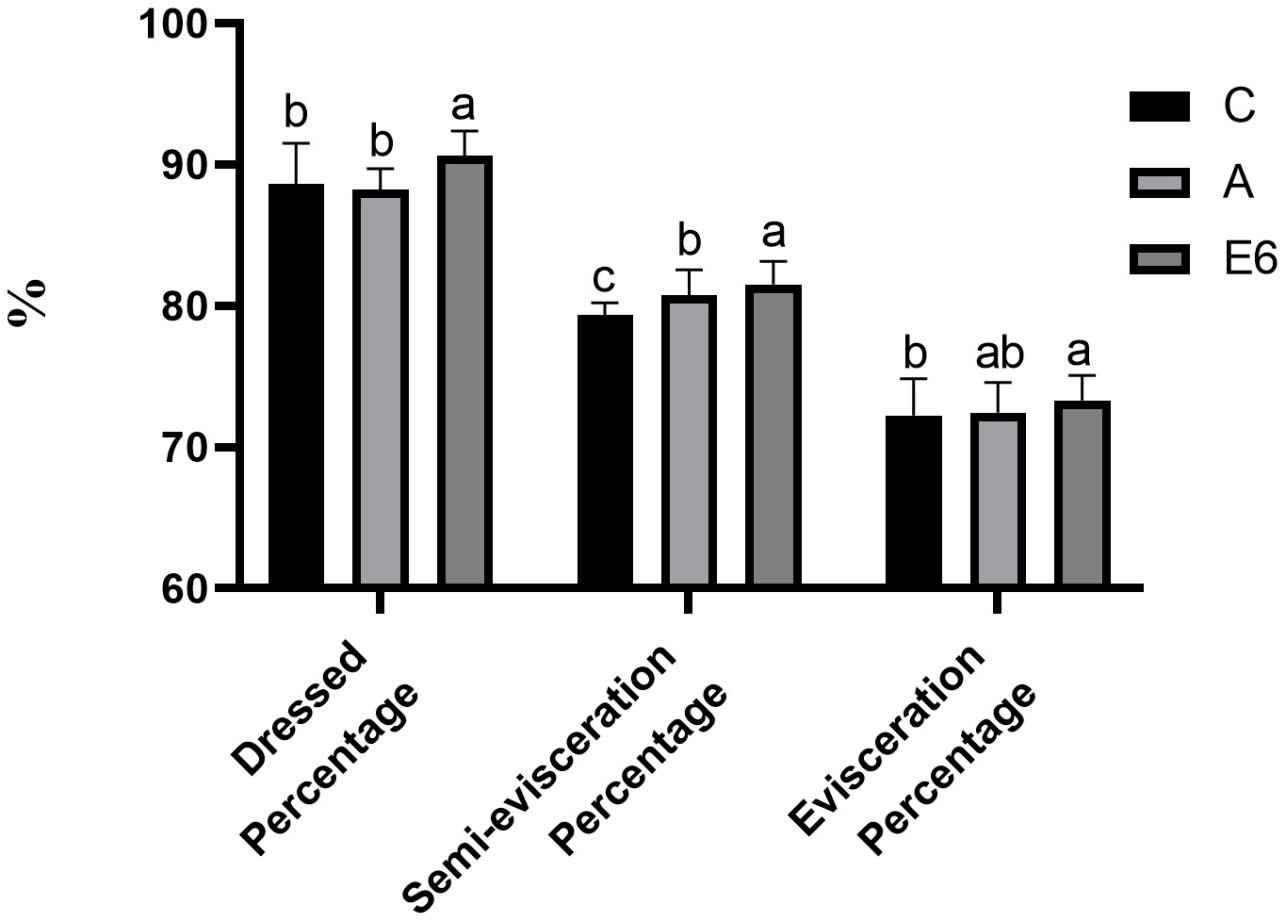
Effects of synbiotics enzyme complex on the dressed, semi-evisceration and evisceration percentages.

**Figure 3:**
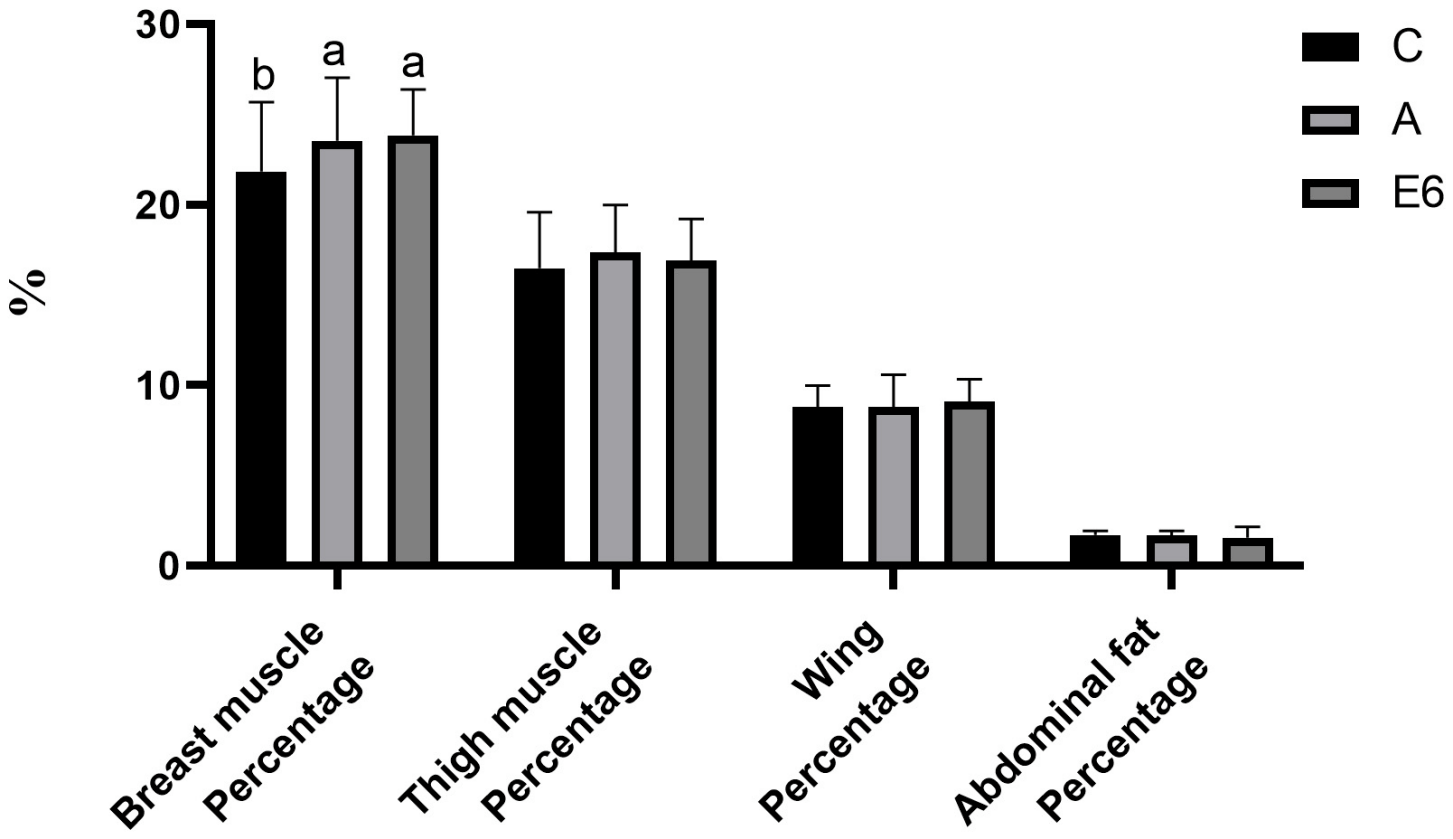
Effects of synbiotics enzyme complex on the breast muscle, thigh muscle, wing and abdominal fat percentages.

The broiler supplementation with SEC significantly (*P* < 0.05) increased the dressed percentage and semi-evisceration percentage (Figure 2) compared to both the control and antibiotic groups. There were no differences (*P* > 0.05) in the percentages of evisceration (Figure 2) and breast muscle (Figure 3) between the SEC treatment group and the antibiotic groups. However, there were notable differences (*P* < 0.05) between the treatment and the control group. Nonetheless, we observed no significant (*P* > 0.05) differences in thigh muscle percentage, wing percentage, or abdomen fat percentage between the treatment groups and the control and antibiotic groups (Figure 3).

### 3.2 Meat quality of the breast and thigh muscles

Table 5 shows the effects of supplementing broilers with SEC on the meat quality characteristics of the breast and thigh muscles. Supplementation of broilers with SEC led to a reduction in the lightness of the breast muscle compared to the control (*P* < 0.05) and the antibiotics group (*P* > 0.05). However, while SEC supplementation led to a significant (*P* < 0.05) reduction in the pH of the breast muscle, it did not affect the redness, yellowness, and shear force of the breast muscle.

**Table 5:** Effects of synbiotics-enzyme complex on meat quality of broilers.

**Muscle**	**Trait**	**Control group**	**Antibiotics group**	**E6 group**
Breast muscle	L	56.85 ± 1.95^a^	54.95 ± 3.78^ab^	53.64 ± 3.65^b^
	a	14.26 ± 2.87	14.96 ± 2.04	14.91 ± 2.93
	b	18.66 ± 3.04	18.92 ± 2.64	17.82 ± 2.85
	pH	5.93 ± 0.24^b^	6.11 ± 0.29^a^	5.91 ± 0.20^b^
	Shear force	17.77 ± 3.60	17.15 ± 1.25	16.35 ± 2.58
Thigh muscle	L	57.59 ± 4.45^a^	54.45 ± 3.35^b^	55.89 ± 4.55^ab^
	a	15.61 ± 2.08	17.39 ± 2.33	15.80 ± 2.86
	b	17.27 ± 3.46	16.83 ± 3.20	16.40 ± 3.53
	pH_24h_	6.23 ± 0.15^c^	6.52 ± 0.22^a^	6.38 ± 0.17^b^
	Shear force	11.41 ± 1.09	11.24 ± 2.61	10.84 ± 0.94

Data in the same column with different shoulder-marked letters indicate significant differences (*P* < 0.05), while the same letters or no letters indicate non-significant differences (*P* > 0.05).

In the thigh muscle, SEC supplementation did not affect the redness, yellowness, and shear force but significantly (*P* < 0.05) affected the lightness and pH after 24 h. The yellowness of the thigh muscle was lower but higher than the control and antibiotics group, respectively. The pH of the thigh muscle of the SEC group was significantly (*P* < 0.05) lower than the control and antibiotics group, respectively.

### 3.3 Small-intestine morphology

Table 6 displays the effects of SEC supplementation on broilers' small intestine morphology. Supplementation of broilers with SEC resulted in significant (*P* < 0.05) increases in the villus height and villus-to-crypt depth ratio (V/C) in comparison to the control and antibiotics group in the duodenum, jejunum, and ileum respectively. Furthermore, in the duodenum, the SEC treatment group had significantly (*P* < 0.05) lower crypt depth than the antibiotics group but was insignificantly (*P* > 0.05) lower than the control group. In the jejunum and ileum, supplementing the broilers with SEC led to a significant (*P* < 0.05) reduction in crypt depth compared to the control and antibiotic groups.

**Table 6:** Effects of synbiotics-enzyme complex on intestinal morphology.

**Part**	**Index**	**Group**		
		**C**	**A**	**E6**
Duodenum	VILLUS height	1,527.64 ± 52.17^c^	1,621.85 ± 45.24^b^	1,768.37 ± 50.94^a^
	Crypt depth	190.64 ± 4.17^b^	196.25 ± 5.12^a^	190.24 ± 6.89^b^
	**V/C**	8.01 ± 0.20^c^	8.27 ± 0.22^b^	9.30 ± 0.29^a^
Jejunum	Villus height	992.25 ± 50.06^c^	1,048.40 ± 50.32^b^	1,130.17 ± 40.48^a^
	Crypt depth	146.36 ± 1.40^b^	148.33 ± 1.92^a^	139.13 ± 1.86^c^
	**V/C**	6.78 ± 0.34^c^	7.07 ± 0.27^b^	8.12 ± 0.20^a^
Ileum	Villus height	786.31 ± 37.29^c^	824.74 ± 28.26^b^	945.21 ± 29.22^a^
	Crypt depth	157.53 ± 6.63^a^	158.24 ± 4.83^a^	152.25 ± 3.20^b^
	**V/C**	5.01 ± 0.42^b^	5.22 ± 0.29^b^	6.21 ± 0.31^a^

Data in the same column with different shoulder-marked letters indicate significant differences (*P* < 0.05), while the same letters or no letters indicate non-significant differences (*P* > 0.05).

### 3.4 Immune organ index

**Figure 4:**
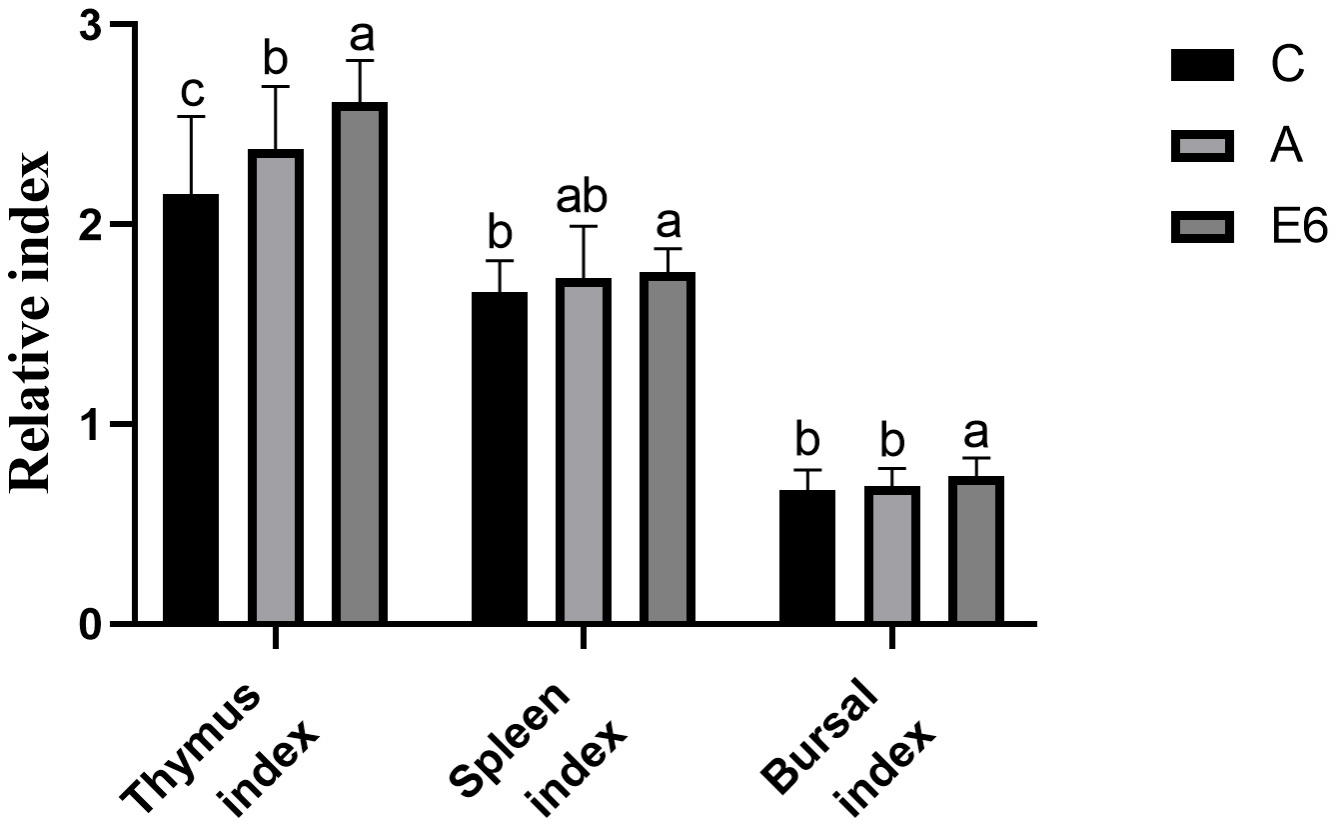
Effects of synbiotics enzyme complex on Immune organ sizes.

Figure 4 shows the effects of SEC supplementation on the broiler's immune organ indexes. The spleen, bursal, and thymus indexes increased significantly (*P* < 0.05) with the addition of SEC in broiler diets. The SEC-supplemented group had significantly (*P* < 0.05) higher spleen, index bursal, and thymus than the control and antibiotic groups indexes.

## 4 Discussion

Average daily feed intake (ADFI), average daily gain (ADG), and feed-to-gain ratio (F/G), which are important indicators for measuring chicken production performance, have a direct impact on the economic efficiency of broiler farms. The results of this study revealed that adding synbiotic-enzyme complex (SEC) to broilers' diets significantly improved the ADG and F/G. The 0.1% SEC inclusion level performed better than the control group, the antibiotics group, and the other inclusion levels. Supplementation with SEC also affected the broilers' body conformation by significantly increasing body slope length, chest width, chest depth, keel length, and shank circumference. Yang et al. (18) reported increased ADG in broilers fed *Clostridium butyricum*, a component of SEC in this study. Other studies documented also support the findings of this study. Ren et al. (7), just as in this study, reported a decreased F/G in broilers fed diets with *Bacillus subtilis*, also a component of SEC. Feeding broilers with another component of this SEC, *mannose oligosaccharides*, also improved the ADFI, ADG, and F/G (19). ADG of broilers was improved in broilers fed with *glucose oxidase* compared to the control chickens (12). Dietary supplementation *of alpha-galactosidase* enhanced growth performance in broiler chickens (11). Furthermore, Dar et al. (20) also reported increased body measurements in cross-bred calves fed Synbiotics.

Synbiotics can boost health and body weight by stimulating the action of one or more bacteria in the large intestine (21), and this may lead to improvements in the body weight and conformation of the broilers. Just as in this study, the synbiotic application of prebiotics, probiotics, and enzymes improved the overall performance of broilers in other studies (22–24). Additional studies indicate that administering synbiotics to broiler chickens stimulates the release of several enzymes (such as proteolytic, lipolytic enzymes, and amylolytic) in the chicken intestine by probiotic microorganisms, hence aiding in the digestion of nutrients (15). This may also be the reason for the increased growth performance and conformation of broilers fed synbiotics-enzyme complex in this study.

The slaughter percentage, breast muscle percentage, wing percentage, and leg muscle percentages are essential measures of broilers' performance and serve as a valuable assessment of carcass characteristics. Feeding broilers with SEC at 0.1% in this study increased the carcass percentage, semi-evisceration percentage, evisceration percentage, and breast muscle percentage compared to the control and the antibiotics group. Furthermore, compared to the control, the treatment group had insignificantly higher thigh muscle and wing percentages and insignificantly reduced abdominal fat percentages. Abdel-Raheem and Sherief Abd-Allah (9) reported a similar finding where the broilers fed synbiotics had better carcass yield than those fed single probiotics and the control. However, Zou et al. (22) reported no significant differences in carcass traits between the control groups and the groups fed multi-strain probiotics. The differences may be attributed to differences in basal diets fed to these birds.

The physical meat quality traits, such as pH, color, and tenderness, are crucial in determining meat products' acceptability, storage, and processability. Glycogen breakdown results in the buildup of lactic acid in the muscles, which causes a decrease in pH after 24 h (25). There is an association between the pH value with color, cooking loss, tenderness, shelf-life, and other characteristics (26). This study revealed that supplementation of broilers with SEC at 0.1% led to a significantly reduced pH after 24 h compared to the antibiotics group but was insignificantly lower than the control group. However, the SEC inclusion significantly increased the pH of the thigh muscle more than in the control group but less than in the antibiotic group. Nevertheless, supplementation of the SEC did not significantly affect the lightness, redness, and yellowness in both the breast and thigh muscles between the control and treatment groups in direct contrast to the findings of (27). Gurram et al. (28) reported similar results when they fed broilers with different combinations of probiotics, chicory root powder, and coriander seed powder. Zou et al. (22) reported similar findings in broilers fed multi-strain probiotics. For most broiler producers and breeders, the goal is to have chickens with increased carcass yield and breast size (26). This study established that SEC improved the carcass yield, breast muscle yield and quality.

The immune organ index, the ratio of immune organs to live body weight, indicates the animal's immune functional status. The relative weight of the spleen, thymus, and bursa are closely connected to the immune response (29). Increased weight of lymphoid organs (bursa, spleen, and thymus) also enhances the immune response, both specific and non-specific, by activating macrophages, boosting cytokine production by intraepithelial lymphocytes, and raising immunoglobulin levels (30). The growth, development, and division of immune cells may contribute to the increase in the weight of immune organs in broilers (31). The current study established that supplementation with SEC at 0.1% leads to a significant increase in the relative weight of the spleen, bursa, and thymus. A tri-strain probiotics comprised of *Clostridium butyricum, Bacillus subtilis*, and *Lactobacillus acidophilus* significantly increased the bursal index in broilers (27). Furthermore, the probiotic and coriander combination also led to increased bursa and spleen weight (28). However, Zou et al. (22) also reported a significantly increased spleen index and a non-significant increase in thymus and bursa index after feeding male broilers with a multi-strain probiotic. Taken together with those of other studies, the findings of this study suggest that SEC inclusion at 0.1% could help enhance the immunity of broilers and work as an alternative to antibiotics.

Increased villi length indicates a larger surface area leading to enhanced adsorption capacity (32, 33). However, shorter villi and deeper crypts can impair nutrient absorption, heightened secretions in the gastrointestinal tract, and reduced performance (34). There is an association between shorter villi length and visible abnormalities that indicate dysbacteriosis (35). To gain a deeper comprehension of how the SEC in this study influences and regulates nutrient digestion and absorption, we measured small intestinal development. This study revealed that supplementation of the SEC at 0.1% increased the villus height and significantly increased the V/C ratio in the duodenum, jejunum, and ileum. This indicated that supplementing the SEC in this study led to improved digestion and absorption capacity. This justifies the higher ADG, improved F/G, and increased body morphometry in the challenged broilers. Feeding broilers with *Bacillus subtilis* combined with *serratiopeptidase*, a proteolytic enzyme, also led to increased villus height and reduced crypt depth (24). The current research results align with the discoveries made by Ali et al. (36), who determined that adding probiotics notably raised the jejunal and duodenal villus length to crypt depth ratio, leading to improved nutritional absorption. The current study's findings also align with the results of Feng et al. (37) and Sun et al. (38), who showed that adding probiotics and enzymes to broilers' diets enhanced the ratio of villus height to crypt depth in their small intestine. These studies taken together with the current study provide evidence that SEC at 0.1% could help improve nutrient digestion in broilers.

## 5 Conclusion

In conclusion, supplementing broilers with a symbiotic enzyme complex (SEC) containing prebiotics (*mannose oligosaccharides*), probiotics (*Clostridium butyricum* and *Bacillus subtilis*) and enzymes [*glucose oxidase* (GOD), and α*-galactosidase* (AlphaGal)] at 0.1% leads to increased ADFI, ADG, reduce F/G ratio, increase body morphometry, increased meat quality traits and carcass quality characteristics, improved small intestine functions and size of immune organs. Therefore, this SEC can be used as a replacement for antibiotics and growth promoters in broilers.

## Data Availability

The original contributions presented in the study are included in the article/supplementary material, further inquiries can be directed to the corresponding authors.
